# Pulpotomy versus pulpectomy in the treatment of vital pulp exposure in primary incisors. A systematic review and meta-analysis.

**DOI:** 10.12688/f1000research.16142.3

**Published:** 2019-06-25

**Authors:** Lamia Gadallah, Mahmoud Hamdy, Adel El Bardissy, Mohamed Abou El Yazeed

**Affiliations:** 1 Orthodontics and Pediatric Dentistry Department, National Research Centre, Egypt, Nasr city, Cairo, 11727, Egypt; 2 Pediatric Dentistry and Dental Public Health, Faculty of Dentistry, Cairo University, Cairo, Cairo, Egypt

**Keywords:** Pulpotomy, Pulpectomy, Root Canal Therapy, Primary Incisor, Vital Pulp Exposure

## Abstract

**Background**: Early childhood caries is a serious public health problem. When caries extend to involve the pulp, various forms of pulp treatment are tried to stimulate tooth repair. Although pulpotomy is the treatment of choice for vital primary tooth pulp exposure but there is a trend among many dentists to perform pulpectomies  in vital primary incisors. This study aimed to assess the effect of pulpotomy and pulpectomy in treatment of carious vital pulp exposure in primary incisors.

**Methods:** We searched Pubmed and Cochrane library databases up to March, 2018, OpenGrey for grey literature and ClinicalTrials.gov for ongoing trials. Randomized controlled trials were included and assessed with Cochrane risk of bias tool . Primary outcomes were clinical failure and radiological failure. The effect sizes were calculated as risk ratios with 95%CI using the Mantel-Haenszel method.

**Results:** Four trials were identified for qualitative assessment, only three trials were included in meta-analysis after exclusion of one trial due to its high risk of bias. The pooled results of the longest follow up period for clinical failure showed no statistically significant difference between pulpotomy and pulpectomy. The relative risk (RR) was e 2.69, 95% CI 0.76 to 9.58 for clinical failure. For radiographic failure, the sensitivity analysis showed RR 0.45, 95% CI 0.25 to 0.83 with a higher risk for radiographic failure in pulpectomy. The evidence was limited by the small number of trials included in the meta-analysis.

**Conclusions:** Both pulpotomy and pulpectomy can be used successfully in the treatment of vital pulp exposure in primary incisors. Further high quality studies comparing between pulpotomy and pulpectomy in primary incisors with longer follow up period till exfoliation time are needed.

## Introduction

Dental caries is an international public health challenge, especially among young children. Early childhood caries (ECC) is a serious public health problem in both developing and industrialized countries ^1^. ECC has been considered to be at epidemic proportions in the developing world ^2– 6^. Treatment of ECC can be accomplished through different types of intervention. When caries extend to involve the pulp, various forms of pulp treatment have been used to treat and/or remove the pulp or to stimulate tooth repair. The choice of technique is as important as the choice between the different pharmacotherapeutic agents which are used in the treatment of primary teeth ^7^.

Although pulpotomy is the treatment of choice for vital primary tooth pulp exposure throughout the pediatric dental literature ^8^, the current trend amongst many dentists is to perform pulpectomies for the pulp treatment of carious vital primary anterior teeth ^9^.

The most common materials used for pulpotomy are formocresol, ferric sulfate, also calcium hydroxide has been used, but with less long term success and more recently mineral trioxide aggregate (MTA) which is much more expensive ^8, 10^.

According to the latest recommendations of the American Academy of Pediatric Dentistry, both formocresol and MTA are strongly recommended to be used in pulpotomy with moderate quality of evidence. Ferric sulfate, laser and sodium hypochlorite are conditionally recommended but as for calcium hydroxide there is a recommendation against its use in pulpotomy ^11^.

In pulpectomy, a resorbable material such as nonreinforced zinc/oxide eugenol (ZOE), a combination paste of iodoform and calcium hydroxide (Vitapex, Metapex) or a combination paste of zinc oxide and eugenol, iodoform and calcium hydroxide (Endoflas) are used to fill the canals ^12^.

According to a Cochrane systematic review, there was no conclusive evidence supporting the superiority of one material for use in pulpectomy. Zinc oxide and eugenol, Metapex and Endoflas were found to be equally effective while with low quality of evidence zinc oxide and eugenol may be better than Vitapex but further research is required for confirmation ^13^.

There are few studies that have compared pulpectomies with pulpotomies in vital primary incisors ^14^. Therefore the claim that pulpotomies don’t work in primary anterior teeth is not supported by high-quality evidence from research. Moreover two studies recently showed that there was no significant difference in success rates of pulpotomies and pulpectomies in the pulp treatment of asymptomatic vital primary incisors ^9, 15^.

We therefore aimed to determine in patients with carious vital pulp exposure in primary incisors if pulpotomy is better than pulpectomy in terms of pain, soft tissue pathology, tooth mobility, pathological root resorption, periapical radiolucency, pulp canal obliteration and tooth survival based on evidence from randomized controlled trials.

## Methods

### Criteria for considering studies for this review

***Types of participants.*** Children with carious vital pulp exposure in primary incisors.

***Types of interventions.*** Pulpotomy and pulpectomy (root canal treatment) techniques with different medicaments.

### Types of outcome measures

***Primary outcomes.*** We defined two primary outcomes: clinical failure and radiological failure.

***Secondary outcomes.*** According to the core set of component outcomes as specified by Smaïl-Faugeron *et al*. ^16^ these secondary outcomes were considered:

Secondary clinical outcomes: pain, soft tissue pathology (gingival swelling, fistulous tract), pathological mobility and tooth survival.

Secondary radiographical outcomes: pathological radiolucency, pathological root resorption, pulp canal obliteration.

### Types of studies

Randomized controlled trials comparing pulpotomy and pulpectomy techniques in the treatment of carious vital pulp exposure in primary incisors were included.

### Search methods

***Electronic search.*** We searched the electronic databases as the Cochrane library to 1/3/2018 and Pubmed to 1/3/2018. We developed detailed search strategies for each database searched. We placed no restrictions on the date of publication when searching the electronic databases. The search strategy included appropriate keywords, and Mesh terms when applicable; combined with Boolean operators “AND”, “OR” and “NOT as shown in Table 1 and Table 2. We also searched OpenGrey for grey literature and ClinicalTrials.gov for ongoing trials.

**Table 1.** Index terms used in search with synonyms.

PICO item	item	Synonyms
**P**	Patients with carious vital pulp exposure in primary anterior teeth	Primary teeth (tooth) Deciduous teeth (tooth) Milky teeth (tooth) Baby teeth (tooth) Incisor(s) Anterior teeth (tooth) Vital teeth (tooth) Pulpally exposed teeth (tooth)
**I**	pulpotomy	Pulpotomy Pulpotomies Vital pulp therapy Dental pulp exposure
**C**	pulpectomy	Pulpectomy Pulpectomies Root canal therapy

**Table 2.** Detailed search strategy for Pubmed and Cochrane library.

	item	Pubmed 11/6	Cochrane 11/6
#1	Tooth, deciduous (Mesh Term)	10215	411
#2	Incisor	22967	1012
#3	Incisors	28128	
#4	Anterior teeth	11087	700
#5	Anterior tooth	8907	
#6	Vital teeth	2445	339
#7	Vital tooth	1923	
#8	Pulpally exposed teeth	5	4
#9	Pulpally exposed tooth	4	
#10	#2 OR #3 OR #4 OR #5	34393	1495
#11	#6 OR #7 OR #8 OR #9	2449	341
#12	#1 AND # 10 AND #11	22	6
#13	Pulpotomy	1416	152
#14	Pulpotomies	1442	
#15	Vital pulp therapy	722	88
#16	Dental pulp exposure	1564	120
#17	#13 OR #14 OR #15 OR # 16	331	282
#18	#12 AND #17	11	4
#19	Pulpectomy	1277	117
#20	Pulpectomies	1291	
#21	Root canal therapy	18604	696
#22	#19 OR #20 OR # 21	19281	779
#23	#12 AND #22	8	4
#24	#18 AND #23	13	4

***Hand searching.*** We hand searched the reference lists of the included full text articles.

### Data collection and analysis

***Selection of studies.*** Two review authors (LG and AEB).independently scanned the titles of all reports identified by the search to determine whether the studies were relevant. They independently scanned selected abstracts to determine whether the study was relevant. After obtaining the full report for all relevant articles, they independently scanned the full reports and completed the data extraction form to determine whether the article should be included or excluded. Disagreements at each stage were resolved by discussion. If this did not resolve the disagreement a third author was invited to settle the disagreement. This did not occur over the course of this study.

***Data extraction and management.*** Two review authors (LG and AEB) collected the data independently using a specially designed data extraction form ( Dataset 1 ^17^).

For each trial, the following data were recorded: the year of publication, the country where the trial took place, detailed description of methodology, sample size, mean age of participants, duration of follow-up and reported outcomes. We contacted the authors of randomized controlled trials for missing information if needed.

***Assessment of risk of bias in included studies.*** Two review authors (LG and AEB) independently assessed the risk of bias in the included trials. The assessment was according to Cochrane risk of bias tool for quality assessment of randomized controlled trials described in the Cochrane Handbook for Systematic Reviews of Interventions 5.1.0 (updated March 2011) ^18^. Any disagreements between the two authors were resolved by discussion. If this did not resolve the disagreement a third author was invited to settle the disagreement. This did not occur over the course of this study.

***Measures of treatment effect.*** Estimated effect size was calculated as risk ratios with 95% Confidence Interval (CI) for dichotomous outcomes. The unit of analysis was the tooth, because teeth were randomly assigned to intervention.

***Data synthesis.*** The effect sizes and associated 95% confidence intervals were calculated using the Mantel-Haenszel method. If the results from trials were homogenous then fixed effect model was preferred. The statistical analysis was performed with the Review Manager program v.5.3 (RevMan) ^19^.

## Results

**Figure 1.**
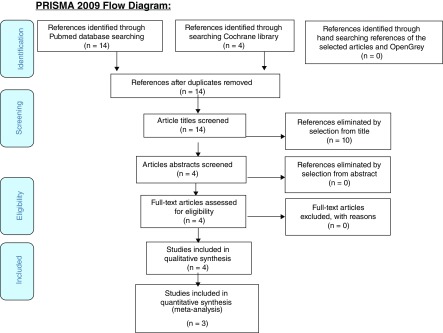
PRISMA Flow diagram.

By searching the different databases, 14 references were identified after removal of the duplicates. By scanning the titles and abstracts, 4 studies were included as shown in the Prisma flow diagram Figure 1 and data extraction was performed (completed PRISMA checklist is available as Supplementary File 1).

A list of included articles is shown in Table 3.

**Table 3.** List of included articles.

Nguyen *et al*. 2017 ^15^	Pubmed
Howley *et al*. 2012 ^9^	Pubmed
Aminabadi *et al*. 2008 ^20^	Pubmed
Casas *et al*. 2004 ^21^	Pubmed

### Study characteristics of included studies

***Year of publication, setting and operators.*** The trials were published between 2004 and 2017. Two trials were in Canada by Nguyen *et al.* ^15^ and Casas *et al.* ^21^ one trial in the United states of America by Howley *et al.* ^9^ and one in Iran by Aminabadi *et al.* ^20^. Operators were dentists in the four trials.

***Participants***. The age range of participants with carious vital pulp exposures in primary incisors varied varying from 18 to 60 months.

***Number of arms***. All four trials by Nguyen *et al.* ^15^, Howley *et al.* ^9^, Aminabadi *et al.* ^20^ and Casas *et al*. ^21^ were two-arm studies.

***Duration of follow up***. Follow up was at 12 and 24 months in two trials, by Aminabadi *et al.* ^20^ and Casas *et al.* ^21^. Follow up was to 23 months at three intervals: 5–9, 10–14, and 15–23 months in the trial by Howley *et al.* ^9^ and at 12 and 18 months in the trial by Nguyen *et al.* ^15^.

***Anesthesia***. Three trials, Nguyen *et al.* ^15^, Howley *et al.* ^9^ and Casas *et al.* ^21^, were under general anesthesia and one trial was under local anesthesia by Aminabadi *et al.* ^20^.

***Rubber dam***. All four trials used rubber dam isolation ^9, 15, 20, 21^.

***Treatment***. The following treatments were compared in the included trials:

The Casas *et al.* trial used ferric sulphate in pulpotomy compared with zinc oxide and eugenol in pulpectomy in 21.

Formocresol in pulpotomy was compared with zinc oxide and eugenol in pulpectomy in the Aminabadi *et al.* trial ^20^.

Formocresol in pulpotomy was compared with vitapex (calcium hydroxide/iodoform paste) in pulpectomy in the Howley *et al.* trial ^9^.

Ferric sulfate and mineral trioxide aggregate in pulpotomy was compared with zinc oxide and eugenol in pulpectomy in the Nguyen *et al.* trial ^15^.

***Medicaments***

**Pulpotomy**

Two trials by Howley *et al.* ^9^ and Aminabadi *et al.* ^20^ used formocresol following hemostasis with a cotton pellet and zinc oxide and eugenol as capping material. One trial used ferric sulphate to achieve hemostasis and zinc oxide and eugenol as capping material, Casas *et al.* ^21^, and one trial used ferric sulfate to achieve hemostasis, with mineral trioxide aggregate as a capping material, Nguyen *et al.* ^15^.

**Pulpectomy**

Zinc oxide and eugenol was used in three trials by Nguyen *et al.* ^15^, Aminabadi *et al.* ^20^ and Casas *et al.* ^21^. One trial, Howley *et al.,* used vitapex (calcium hydroxide/iodoform paste) ^9^.

***Pulp access***. Following caries removal and pulp exposure, the pulp chamber was accessed using a sterile no. 56 bur in a water-cooled high-speed handpiece and was refined using a sterile round bur in a slow-speed handpiece in three trials, Nguyen *et al.* ^15^, Aminabadi *et al.* ^20^ and Casas *et al.* ^21^.

In the trial by Howley *et al.* ^9^ the pulp chamber was unroofed using a no. 330 sterile bur in a water-cooled high-speed handpiece then the access was refined using a sterile round bur in a slow-speed handpiece.

***Pulpotomy.*** The coronal pulp was amputated using a sharp spoon excavator in two trials by Howley *et al.* ^9^ and Aminabadi *et al.* ^20^ and was amputated using a sterile low-speed round bur in another two trials by Nguyen *et al.* ^15^ and Casas *et al.* ^21^.

***Root canal treatment.*** In three trials Nguyen *et al.* ^15^, Howley *et al.* ^9^ and Casas *et al*. ^21^ pulp tissue was removed *en bloc* using two or more endodontic files (Hedström ﬁles or K files), if the first attempt was unsuccessful, the procedure was repeated until all of the pulp tissue was removed.

In the fourth trial by Aminabadi *et al.* ^20^ an endodontic K file was introduced to the working length after a periapical radiograph was taken, and most of the pulp tissue was removed completely on the first attempt. If the first attempt was unsuccessful, the procedure was repeated and canals were generally enlarged three sizes past the initial file.

***Irrigation***. The irrigation solution was sterile water in Nguyen *et al*. trial ^15^ and saline in the trials by Howley *et al.* ^9^ and Aminabadi *et al.* ^20^ while in the fourth trial by Casas *et al.* ^21^ the irrigating solution was unidentified.

***Final restoration***. Resin restorations was performed in three trials, Nguyen *et al.* ^15^, Aminabadi *et al.* ^20^ and Casas *et al.* ^21^. Full coverage crown whether stainless steel crown (SCC) or SCC with white esthetic veneer were used in Howley *et al. trial* ^9^.

***Number of visits***. In the four trials by Nguyen *et al.* ^15^, Howley *et al.* ^9^, Aminabadi *et al.* ^20^ and Casas *et al.* ^21^ the intervention was completed in one session, whether they were performed under general or local anesthesia.

### Results of studies

***Clinical failure***. Clinical failure was reported to be 2% and 3% for pulpotomy at 12 months and 18 months respectively, while for the pulpectomy there were none at 12 months, and 1% at the 18 months follow up period in the Nguyen *et al.* trial ^15^. No clinical failures were reported for neither pulpotomy nor pulpectomy in this 23 months trial by Howley *et al.* ^9^. The clinical failure rate was 13.1% for pulpotomy and 4.4% for pulpectomy at 2 years follow up for Aminabadi *et al.* ^20^. Clinical failure was 22% in the pulpotomy group, with no clinical failures in the pulpectomy group in the trial by Casas *et al.* ^21^.

***Radiological failure***. Radiographical failure was reported to be 3% and 7% in the pulpotomy group at 12 and 18 months respectively, while for the pulpectomy group it was 8% at both 12 months and 18 months follow ups in the Nguyen *et al.* trial ^15^. Cumulative radiographical results showed failure in 11% in the pulpotomy group, and 27% in pulpectomy group in the Howley *et al.* trial ^9^. Radiographical failure was 23.9% in the pulpotomy group, and 8.6% in pulpectomy group at 2 years follow up as reported by Aminabadi *et al.* ^20^. Radiographical failure was 41% in the pulpotomy group and 18% in pulpectomy group at 2 years follow up trial by Casas *et al.* ^21^.

***Overall failure***. One incisor was lost early and counted as a failure in the pulpotomy group in the Howley *et al.* trial ^9^. One tooth had to be extracted postoperatively (2.2%) in pulpotomy group in the Aminabadi *et al.* trial ^20^.

***Pain***. One tooth with spontaneous pain (1%) was reported in pulpotomy group in Nguyen *et al.* trial ^15^. No pain was reported in either group in Howley *et al.* trial ^9^. Two teeth were reported as having spontaneous pain (4.4%) in the pulpotomy group, and one (2.2%) in the pulpectomy group in Aminabadi *et al*. ^20^. No pain was reported in either group in the Casas *et al.* trial ^21^.

***Soft tissue pathology***. Two teeth showed fistula (2%) in the pulpotomy group, and one tooth had soft tissue swelling (1%) in pulpectomy group in the Nguyen *et al.* trial ^15^. No soft tissue pathology was reported in either group in Howley *et al.* ^9^. The presence of fistula was reported in 3 teeth (6.6%) in the pulpotomy group of the Aminabadi *et al.* trial ^20^. The presence of gingival swelling or parulis in 9 teeth (22%) in pulpotomy group was reported by Casas *et al.* ^21^.

***Pathological mobility***. Pathologic mobility was not reported for any tooth in three trials, Howley *et al.* ^9^ Aminabadi *et al.* ^20^ and Casas *et al.* ^21^. Only one tooth with pathological mobility was reported by Nguyen *et al.* ^15^.

***Pathological radiolucency***. The odds ratio for periapical radiolucency was 177.55; 95% CI 20.29 to 1554.01 (P<0.0001; chi-square test) in the Nguyen *et al.* trial ^15^. After 23 months follow up, 7 teeth (23%) showed frank periapical radiolucency in the pulpectomy group, while only 1 tooth (3%) showed frank periapical radiolucency in the pulpotomy group in Howley *et al.* trial ^9^. At 2 years follow up, 5 teeth (11.11%) showed periapical radiolucency in the pulpotomy group, while only one tooth (2.17%) in the pulpectomy group showed periapical radiolucency in the Aminabadi *et al.* trial ^20^. At 2 years follow up, 7 teeth (58%) showed periapical radiolucency in the pulpotomy group, and 3 teeth (27%) in the pulpectomy group in Casas *et al.* ^21^.

***Pathological root resorption***. The odds ratio for external root resorption was 136.41;95% CI 15.02 to 1238.27 (P<0.0001; chi-square test) in Nguyen *et al.* trial ^15^. After 23 months follow up, 2 teeth (7%) showed large external root resorption in the pulpotomy group, and 4 teeth (14%) in the pulpectomy group, while for internal resorption, one tooth (3%) showed perforating internal root resorption in the pulpotomy group in the Howley *et al.* trial ^9^.

At 2 years follow up, pathologic external or internal root resorption occurred in 6 teeth (13.3%) of the pulpotomy group and in 2 teeth (4.34%) of the pulpectomy group in the Aminabadi *et al.* trial ^20^.

At 2 years follow up pathologic external root resorption occured in 4 teeth (33%) in the pulpotomy group and in 3 teeth (27%) in the pulpectomy group, internal resorption was observed in 17% of the pulpotomy group in Casas *et al.* trial ^21^.

***Pulp canal obliteration***. At 23 months, pulp canal obliteration was seen in 18 teeth (60%) in the pulpotomy group in Howley *et al.* ^9^. At 2 year follow up, no teeth showed pulp canal obliteration in Aminabadi *et al.* trial ^20^. At 2 years follow up, 3 teeth (25%) showed pulp canal obliteration in the pulpotomy group in the Casas *et al.* trial ^21^.

***Tooth survival***. The survival rate was 0.94 (95 % CI equals 0.89 to 0.97) for pulpotomy and 0.97 for pulpectomy at 18 months in Nguyen *et al.* trial ^15^. The survival rate was 63% for pulpotomy and 85% for pulpectomy at 2 years follow up in Casas *et al.* trial ^21^.

**Figure 2.**
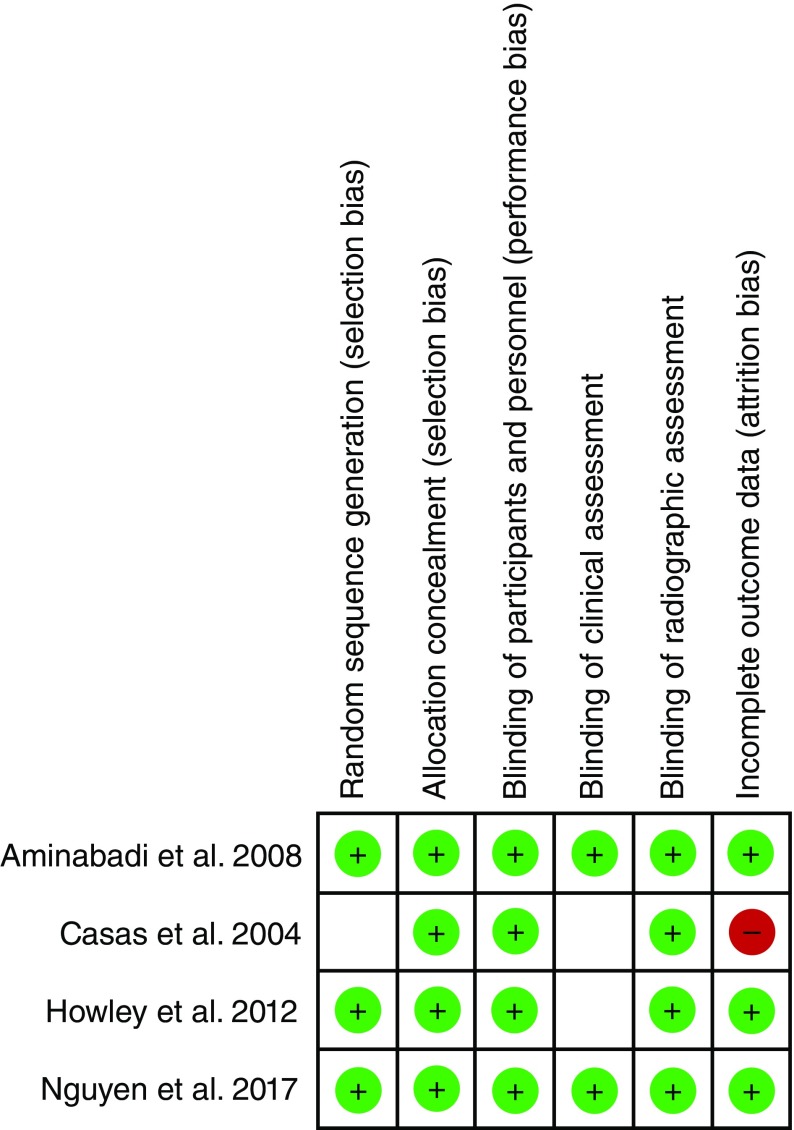
Risk of bias summary of included studies.

***Risk of bias in included studies***. The risk of bias of included studies is shown in Table 4, Table 5, Table 6, and Table 7. The overall risk of bias was low in two trials, Nguyen *et al.* ^15^ and Aminabadi *et al.* ^20^. The risk of bias was unclear for clinical assessment in two trials, Howley *et al.* ^9^ and Casas *et al.* ^21^, and also for random sequence generation in Casas *et al.* ^21^. One trial, Casas *et al.* ^21^, showed high risk of bias due to high percentage of dropped out cases. The risk of bias of included studies is also summarized in Figure 2.

**Table 4.** Risk of bias in Nguyen *et al*. 2017.

Nguyen *et al*. 2017 ^15^		
Bias	Authors' judgement	Support for judgement
Random sequence generation (selection bias)	Low risk	Computer-generated simple random numbers sequence with a one to one allocation ratio.
Allocation concealment (selection bias)	Low risk	Quote: Allocation occurred after induction of general anesthesia to ensure allocation concealment. The pediatric dentist, nurse, or assistant directed subjects at the time of dental surgery to the appropriate treatment group they had been assigned to by the investigator.
Blinding of participants and personnel (performance bias)	Low risk	Quote: All other contributors were blinded to generation and implementation of the treatment assignment
Blinding of clinical outcomes assessment	Low risk	Quote: A single investigator, who did not perform any pulp therapy or participate in radiographic evaluation, performed all clinical assessments.
Blinding of radiological outcomes assessment	Low risk	Quote: Two experienced pediatric dentists who did not participate in protocol development or treatment performed radiographic assessments. It is not possible to blind the assessors due to the nature of treatment received.
Incomplete outcome data (attrition bias)	Low risk	9% drop out of 172 incisors at 12 months, due to loss to follow-up (n=13) and due to trauma (n=2). 21% drop out of 172 incisors at 18 months, due to loss to follow-up or dropout (n =31), exfoliation (n = 3), and trauma (n = 3).
Reporting bias	Low risk	We revised the protocol that was registered with the ClinicalTrials.gov Protocol and Registration System (ID no. NCT02019563)

**Table 5.** Risk of bias in Howley *et al*. 2012.

Howley *et al*. 2012 ^9^		
Bias	Authors' judgement	Support for judgement
Random sequence generation (selection bias)	Low risk	An incisor in each pair was randomly assigned by a coin toss.
Allocation concealment (selection bias)	Low risk	Quote: An incisor in each pair was randomly assigned, by a coin toss to either the experimental group or the control group with the contralateral- paired incisor being designated to the other treatment group
Blinding of participants and personnel (performance bias)	Low risk	It is not possible to blind the operator and the participant blinding is ineffective on outcomes
Blinding of clinical outcomes assessment	unclear risk	Insufficient information to make a clear judgement
Blinding of radiological outcomes assessment	Low risk	Quote: The radiographs were evaluated independently by 2 standardized and calibrated examiners who were not otherwise involved in the study. It is not possible to blind the assessors due to the nature of treatment received.
Incomplete outcome data (attrition bias)	Low risk	29 study patients, 3 patients failed to return for follow-up.

**Table 6.** Risk of bias in Aminabadi *et al*. 2008.

Aminabadi *et al*. 2008 ^20^		
Bias	Authors' judgement	Support for judgement
Random sequence generation (selection bias)	Low risk	Coin tossing
Allocation concealment (selection bias)	Low risk	Quote: For each patient, by coin tossing, if one tooth was randomly assigned for formocresol pulpotomy then root canal therapy (RCT) was performed on the other incisor.
Blinding of participants and personnel (performance bias)	Low risk	It is not possible to blind the operator and the participant blinding is ineffective on outcomes
Blinding of clinical outcomes assessment	Low risk	Quote: Two clinicians who did not perform any treatments analyzed the clinical and radiographic outcomes
Blinding of radiological outcomes assessment	Low risk	Quote: Two clinicians who did not perform any treatments analyzed the clinical and radiographic outcomes It is not possible to blind the assessors due to the nature of treatment received.
Incomplete outcome data (attrition bias)	Low risk	4 subjects were dropped out of 50 patients.

**Table 7.** Risk of bias in Casas *et al*. 2004.

Casas *et al*. 2004 ^19^		
Bias	Authors' judgement	Support for judgement
Random sequence generation (selection bias)	Unclear risk	Insufficient information to make a clear judgement
Allocation concealment (selection bias)	Low risk	Quote: Quality assurance checks were performed by 1 of the investigators (MAL), who did not provide treatment or review postoperative radiographs, to ensure that the investigators who provided treatment complied with the randomization protocol
Blinding of participants and personnel (performance bias) All outcomes	Low risk	It is not possible to blind the operator and the participant blinding is ineffective on outcomes
Blinding of clinical outcomes assessment	unclear risk	Insufficient information to make a clear judgement.
Blinding of radiological outcomes assessment	Low risk	Quote: Two independent pediatric dentists who were not otherwise involved in the investigation evaluated the radiographs. It is not possible to blind the assessors due to the nature of treatment received.
Incomplete outcome data (attrition bias) All outcomes	High risk	Quote: Of the enrolled participants, 64% returned for at least 1 evaluation. 36% drop out in the pulpotomy group and 48% drop out in the root canal treatment group at 2 years follow up

***Synthesis of results***. Only three trials were included in the meta-analysis by Nguyen *et al.* ^15^ Howley *et al.* ^9^ and Aminabadi *et al.* ^20^ as one trial by Casas *et al.* ^21^ was excluded due to its high risk of bias. Data were extractable from all three RCTs totaling 338 teeth.

The data of the longest follow up period was included in the meta-analysis, this was 24 months for Aminabadi *et al.* ^20^, 18 months for Nguyen *et al.* ^15^, and at 15 to 23 months for Howley *et al.* ^9^.

**Clinical failure**

**Figure 3.**
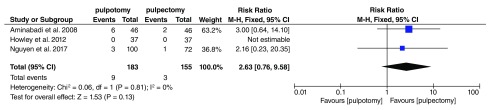
Meta-analysis for clinical failure.

At the longest follow up period the pooled results showed no statistically significant difference in clinical failure for pulpotomy compared to pulpectomy (RR 2.69 ,95% CI 0.76 to 9.58) as shown in Figure 3.

The Howley et al. study was excluded from the meta-analysis of clinical failure as there were no events in both groups.

It is the standard practice for the meta-analysis for risk ratio when there are no events in both arms ^18^.

**Radiographic failure**

**Figure 4.**
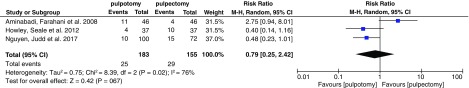
Meta-analysis for radiographic failure.

The pooled results showed RR 0.79, 95% CI 0.46 to 1.21 in radiographical failure for pulpotomy compared to pulpectomy as shown in Figure 4 but considerable heterogeneity was presented in the meta-analysis of radiographic failure due to the presence of one outlying study of Aminabadi *et al*. so we performed the analysis both with and without this study as part of a sensitivity analysis as shown in Table 8.

**Table 8.** Sensitivity analysis for radiographic failure.

	pulpotomy		pulpectomy			Risk Ratio
study or subgroup	Events	Total	Events	Total	Weight	M-H, Random, 95% CI
Howley, Seale *et al*. 2012	4	37	10	37	32.5%	0.40 [0.14, 1.16]
Nguyen, Judd *et al*. 2017	10	100	15	72	67.5%	0.48 [0.23, 1.01]
Total (95% CI)		137		109	100.0%	0.45 [0.25, 0.83]
Total events	14		25			
Heterogeneity: Tau ^2^=0.00; Chi ^2^=0.008, df=1 (P=0.78); I ^2^=0%						
Test for overall effect Z=2.56 (P=0.01)						

The sensitivity analysis for radiographic failure showed RR 0.45, 95% CI 0.25 to 0.83 with statistical significant difference between pulpotomy and pulpectomy and a higher risk for radiographic failure in pulpectomy

**Pain**

The pooled results showed no statistically significant difference in pain for pulpotomy compared to pulpectomy (RR 2.06, 95% CI 0.31 to13.8)).

**Soft tissue pathology**

The pooled results showed no statistically significant difference in soft tissue pathology for pulpotomy compared to pulpectomy (RR 3.11, 95% CI 0.54 to17.7)

**Pathological radiolucency**

The results of two trials showed considerable statistical heterogeneity so no meta-analysis were performed for this outcome.

**Pathological root resorption**

The pooled results of two trials showed no statistically significant difference in pathologic resorption for pulpotomy compared to pulpectomy (RR 1.5, 95% CI 0.56 to 4.04).

Summarized extracted data from included trials Summary data using the study data extraction form Click here for additional data file. Copyright: © 2019 Gadallah L et al. 2019 Data associated with the article are available under the terms of the Creative Commons Zero "No rights reserved" data waiver (CC0 1.0 Public domain dedication).

## Discussion

 Pulp therapy is performed to preserve primary teeth and maintain its developmental, esthetic, and functional capabilities. Pulpotomy and root canal therapy have been both performed as techniques for the management of asymptomatic vital primary incisors with large carious lesions where removal of caries will lead to pulp exposure ^20^. However the preference of many pediatric dentists to perform pulpectomy in primary incisors was due to the fact that they were taught to do so in their pediatric dentistry residencies and not due to evidence from high quality research ^9^. The aim of this systematic review was to compare between pulpotomy and pulpectomy clinically and radiographically in the treatment of carious vital pulp exposure in primary incisors.

Upon performing our systematic search there were only four randomized controlled trials that have compared pulpotomy and pulpectomy outcomes in vital primary incisors ^9, 15, 20, 21^. After exclusion of one trial due to its high risk of bias, only three trials were left to be included in the meta-analysis. The data of the longest follow up period was included as the follow ups were close to each other ranging from 15 months to 24 months and they best reveal the efficacy of the performed techniques.

The results were calculated with risk ratio (RR) effect measure and confidence intervals (CIs). The pooled results of the clinical failure outcome showed the relative risk RR was 2.69 with 95% CI from 0.76 to 9.58, the CI including the number 1 means that there was no statistical significant difference between pulpotomy and pulpectomy cases.

For radiographic failure the RR was 0.79 with 95% CI from 0.25 to 2.42 but considerable statistical heterogeneity was detected due to the presence of one outlying study of Aminabadi *et al*. with results that conflict the other studies so sensitivity analysis was performed that showed that RR was 0.45 with 95% CI 0.25 to 0.83 with higher risk of radiographic failure for pulpectomy rather than pulpotomy.

Although there is no clinical diversity among the included trials that may have led to this statistical heterogeneity, we may relate it to that the criteria of radiographic assessment in Aminabadi *et al*, study was not clearly specified.

The exclusion of a study from a meta-analysis based on their result may introduce bias so the results must be interpreted with an appropriate degree of caution and futher investigaton is required ^18^. For the clinical outcomes pain and soft tissue pathology, the pooled results for these outcomes showed no statistically significant difference between pulpotomy and pulpectomy while pathologic mobility was only reported for one incisor in one trial.

The radiographic outcomes included periapical radiolucency and pathologic root resorption. For pathologic resorption, the pooled results showed no statistically significant difference between pulpotomy and pulpectomy. We considered the tooth to be scored with pathologic root resorption if it showed perforating internal root resorption or large external root resportion while those teeth showing contained internal root resorption or questionable external root resorption were not counted. For periapical radiolucency, only frank radiolucencies were counted and not questionable ones but the results of the trials included was inconsistent.

Pediatric dentists do not consider the radiographic pathological changes as questionable radiolucencies or limited pathological root resorptions to be an absolute indication for extraction taking into consideration the absence of clinical pathological signs or symptoms ^15^.

Overall failure was reported for two pulpotomized incisors in two trials. On the other hand tooth survival although it is an important outcome, but it is not commonly reported.

This study showed that there is no statistical significant difference in clinical success rates of pulpotomy and pulpectomy with different medicaments in the treatment of carious vital pulp exposure in primary incisors while radiographically, pulpectomy may have a higher risk for radiographic failure than pulpotomy and this refutes the misconception among some pediatric dentists that pulpotomy does not work in primary incisors.

The evidence was limited by the small number of trials included in the meta-analysis. The overall risk of bias of primary studies was low for three trials, except for the unclear risk for blinding of clinical assessment which was not effective., We did not have access to all the trial protocols to assess the selective reporting bias except for only one trial.

## Conclusion

There was no statistical significant difference in the clinical success rates of pulpotomies and pulpectomies in the pulp treatment of carious vital pulp exposure in primary incisors while radiographically, pulpectomy may have a higher risk for radiographic failure than pulpotomy. We recommend teaching of pulpotomy as a treatment option for vital pulp exposure in primary incisors in pediatric dentistry residency programs and further high quality studies comparing between pulpotomy and pulpectomy in primary incisors with a longer follow up period till exfoliation time.

## Data availability

The data referenced by this article are under copyright with the following copyright statement: Copyright: © 2019 Gadallah L et al.

Data associated with the article are available under the terms of the Creative Commons Zero "No rights reserved" data waiver (CC0 1.0 Public domain dedication). 

Dataset 1: Summarized extracted data from included trials. 10.5256/f1000research.16142.d218857 ^17^

## Author information

LG is a Researcher Assistant at the Orthodontics and Pediatric Dentistry Department, National Research Centre, Egypt. MH is a Professor at the Pediatric Dentistry and Dental Public Health Department, Faculty of Dentistry, Cairo University, Egypt. AEB is an Associate Professor at the Pediatric Dentistry and Dental Public Health, Faculty of Dentistry, Cairo University, Egypt. MAEY is a Professor at the Orthodontics and Pediatric Dentistry Department, National Research Centre, Egypt.

## Supplementary material

Supplementary File 1: Completed PRISMA checklist.
