# Supplementary material for: Pulpotomy versus pulpectomy in the treatment of vital pulp exposure in primary incisors. A systematic review and meta-analysis
Source: F1000Res. 2019 Jun 25;7:1560. Originally published 2018 Sep 26. [Version 3] doi: 10.12688/f1000research.16142.3 (PMC6584970; doi:10.12688/f1000research.16142.3)
Supplement: Summarized extracted data from included trials — Summary data using the study data extraction form [file f1000research-7-21668-s0000.tgz › ecca2a14-01cb-40e5-be44-544a3a649378_Dataset_1.docx]

**6. Summary of Findings Table (SOFT):**

**A.Baseline and demographic data:**

|  | **Study Design** | **No of patients** | **No of teeth pulpotomy** | **No of teeth pulpectomy** | **Age** | **Gender** | **Country** |
| --- | --- | --- | --- | --- | --- | --- | --- |
| **Nguyen, Judd et al.2017** | Randomized controlled trial | 70 | 100 | 72 | 18-46m  Average:31m | Male:33  Female:37 | Canada. |
| **Howley, Seale et al. 2012** | Randomized controlled trial | 29 | Start with 50  End with 37 | Start with 50  End with 37 | 18-60 m  Mean age:36±10.5 m | Male:17  Female:12 | United states of america |
| **Aminabadi, Farahani et al. 2008** | Randomized controlled trial | 50 | 45 | 46 | 3-4 y | Male:23  Female:27 | Iran |
| **Casas, Kenny et al. 2004** | Clinical trial | 50 | Start with:64  End with:41 | Start with:29  End with:36 | Pulpotomy: 3.3±0.8 y  Pulpectomy: 3.1±0.7 y | Male:29  Female:21 | Canada |

**B.Methodological steps:**

|  | **Conducted by** | **Anesthesia** | **Materials** | **Radiograph** | **Final restoration** | **Follow up** |
| --- | --- | --- | --- | --- | --- | --- |
| **Nguyen, Judd et al.2017** | Five pediatric dentists completed all pulp therapy. Another Two experienced pediatric dentist performed radiographic evaluation. a single investigator bperformed all clinical assessments. | General anesthesia | Pulpotomy:ferric sulfate then mineral trioxide aggregate as a capping material.  Pulpectomy: nonreinforced ZOE |  | acid etch resin | At 12 and 18 months |
| **Howley, Seale et al. 2012** | Two standardized operators completed all treatments and two standardized and calibrated examiners evaluated radiographs | General anesthesia | Pulpotomy:full strength Buckely formcresol and zinc oxide and eugenol as capping material  Pulpectomy: vitapex(calcium hydroxide/iodoform paste) | Standardized maxillary occlusal radiograph | Stainless steel crown(SCC) or SCC with white esthetic veneer | 23m  Mean  (13.0±5.6m)  Intervals:5-9, 10-14, and 15-23 m |
| **Aminabadi, Farahani et al. 2008** | One pediatric dentist completed all treatments and two clinicians analyzed clinical and radiographic outcomes | Local anesthesia | Pulpotomy:formcresol(buckley 1.5%), fortified zinc oxide and eugenol as capping material  Pulpectomy: zinc oxide and eugenol | Size 0 periapical films using bisecting angle technique | Self- cure resin restoration | 24 m  Intervals:12 and 24m |
| **Casas, Kenny et al. 2004** | Three pediatric dentists completed the treatment and two independent pediatric dentists evaluated the radiographs | General anesthesia | Pulpotomy:ferric sulphate and zinc oxide and eugenol as capping material  Pulpectomy:zinc oxide and eugenol | Size 0 periapical films using bisecting angle technique | Acid-etch resin restoration | 2y  Intervals:12 and 24m |

**C.Outcome and results:**

|  | **Outcome** | | | **Results** |  |  |
| --- | --- | --- | --- | --- | --- | --- |
|  | **Name** | **Device** | **unit** | **Pulpotomy** | **Pulpectomy** | **P Value** |
| **Nguyen, Judd et al.2017** | Pathologic root resorption | Radiograph | Binary | Internal  External | External |  |
|  |  |  |  | OR equals 136.41; 95 % CI equals 15.02 to 1238.27 | |  |
|  | Periapical radiolucency |  |  | OR equals 177.55; 95 % CI equals 20.29 to 1554.01 | |  |
|  | Pulp canal obliteration |  |  | OR equals 5.33; 95% CI equals 1.04 to 27.20 |  |  |
|  | Success rate | Radiographical    Clinical |  | At 12m97% At 18m 93%  At 12m98%,at 18m97% | At 12m and 18m 92%  At 12m 100%,18m 99% |  |
| **Howley, Seale et al. 2012** | Pathologic root resorption | Radiograph | Binary | Internal 7%  External 3% | External 10% |  |
|  | Periapical radiolucency | Radiograph | Binary | 3% | 17% |  |
|  | Pulp canal obliteration | Radiograph | Binary | 60% |  |  |
|  | Success rate | Radiographical |  | 89% | 73% | P value: 0.11 |
| **Aminabadi, Farahani et al. 2008** | Pathologic root resorption | Radiograph | Binary | 13.3% | 4.34% |  |
|  | Periapical radiolucency | Radiograph | Binary | 11.11% | 2.17% |  |
|  | Pain | Questionnaire | Binary | 4.4% | 2.2% |  |
|  | Fistulous tract | Visual exam. | Binary | 6.6% |  |  |
|  | Success rate | Clinical &radiographical |  | 86.9%clinical  76.08% radiographical | 95.6%clinical  91.3% radiographical | P value: ˃0.05 clinical  ˂0.05 radiographic |
|  | **Outcome** | **Results** |  |  |  |  |
| ‘ | **Name** | **Device** | **unit** | **Pulpotomy** | **Pulpectomy** | **P Value** |
| **Casas, Kenny et al. 2004** | Pathologic root resorption | Radiograph | Binary | Internal 17%  External 33% | External 27% |  |
|  | Periapical radiolucency | Radiograph | Binary | 58% | 27% |  |
|  | Pulp canal obliteration | Radiograph | Binary | 25% |  |  |
|  | Widening of PDL space | Radiograph | Binary | 67% | 18% |  |
|  | Pain | Questionnaire | Binary | 0% | 0% |  |
|  | Gingival swelling | Visual exam. | Binary | 22% |  |  |
|  | Fistulous tract | Visual exam. | Binary | 22% |  |  |
|  | Success rate | Clinical and radiographical |  | 78%clinical  59%radiographical | 100%clinical  82%radiographical | P value: ˃0.05 radiographical |
