# Supplementary material for: Pulpotomy versus pulpectomy in the treatment of vital pulp exposure in primary incisors. A systematic review and meta-analysis
Source: F1000Res. 2019 Jun 25;7:1560. Originally published 2018 Sep 26. [Version 3] doi: 10.12688/f1000research.16142.3 (PMC6584970; doi:10.12688/f1000research.16142.3)
Supplement: Peer review history

# Peer review: Reviewer response for version 3

**DOI:** 10.5256/f1000research.21668.r50364

**Published:** 2019-07-10

**Authors:** Marcela Baraúna Magno

**Affiliations:** 1 Department of Pediatric Dentistry and Orthodontics, School of Dentistry, Federal University of Rio de Janeiro, Rio de Janeiro, Brazil

The authors performed most of the requested changes. Minor changes are necessary.

**Abstract:**

- Radiographic assessment needs to be added in abstract conclusion.

**Results:**

- According Cochrane, yellow represents unclear. This could be generated in Revman if you justified the unclear classification in software.

I confirm that I have read this submission and believe that I have an appropriate level of expertise to confirm that it is of an acceptable scientific standard.

# Peer review: Reviewer response for version 2

**DOI:** 10.5256/f1000research.19128.r49412

**Published:** 2019-06-19

**Authors:** Marcela Baraúna Magno

**Affiliations:** 1 Department of Pediatric Dentistry and Orthodontics, School of Dentistry, Federal University of Rio de Janeiro, Rio de Janeiro, Brazil

**Abstract:**

- The methodology of bias risk analysis and meta-analysis was lacking.
- If you have word limitation, you can reduce the abstract background.
- Is risk ratio correct or relative risk?
- I was confused. How radiographically did the pulpectomy prove worse?

**Methodology:**

- Differentiate the primary outcome and the secondary outcome. 
- Correct me if I am wrong, but the factors considered in the secondary outcome are those that will determine the primary outcome.
- The search is updated...
- Search is confusing and seems to be wrong.
- I did not understand why at # 22 you crossed # 18 (which would be terms related to 'prior vital teeth' and 'pulpotomy') or terms related to 'pulpectomy' (# 19 or # 20).
- The # 21 was not used at any intersection.
- I did not understand the purpose of # 24.
- If you want studies evaluating pulpotomy X pulpectomy, you should cross terms related to 'anterior teeth' and 'pulpotomy' and 'pulpectomy'.
- If you did not do this kind of crossover, some terms may have been selected wrong. Please review.
- The authors considered homogeneous until the value of i2?

**Results:**

- Include the unclear symbols in figure 2.
- Remove the Howley study, since this is not considered in analysis in figure 3.
- Include a descriptive result for pathological radiolucency. What the studies concluded about this outcome?

**Conclusion: **

- Only for clinical parameters, no?
- Pulpectomy presented more failures than pulpotomy for radiographic parameter.

I confirm that I have read this submission and believe that I have an appropriate level of expertise to confirm that it is of an acceptable scientific standard, however I have significant reservations, as outlined above.

# Response

**Published:** 2019-06-20

**Authors:** Lamia Gadallah

**Affiliations:** National Research Centre, Egypt, Egypt

Dear Dr. Marcela,

We are honored by your reviewing to our article and we took into consideration all your valuable corrections and comments.

Firstly, in the abstract section:

The risk of bias tool and meta analysis method used was added.

The relative risk is called  the risk ratio and both terms are mentioned in Cochrane Handbook for systematic reviews of interventions.

Radiographically the risk ratio value  was 0.45 95%CI 0.25 to 0.83. These values are less than one which means higher risk of radiographic failure in the control(pulpectomy) group.

Secondly, in the methodology section:

The authors chose the primary outcomes according to a clinical point of view that there is a set of outcomes clinically and radiographically that collectively indicate the success or failure of the technique and you are absolutely right that those secondary outcomes are the single outcomes that composed the composite primary outcome, and we preferred to show their effect separately as secondary ones due to their less importance as single outcomes.

Yes the search was last updated in march 2018.

In the search strategy, we thank you for the two mistakes you have highlighted. By revising the saved history of search strategy, we found two editing mistakes, first line #22 is composed of #19,#20 #21 and not #18.

Line 24 is combined with AND not OR because we wanted the studies of incisors and pulpotomy with incisors and pulpectomy, that's how the actual search was.performed, the mistakes were during editing tables.

The I2 is considered homogenous up till 50%. 

Thirdly, the results section:

Figure 2 is according to Risk of bias summary figure in Cochrane Handbook where green represents low risk, red represents high risk and blank is unclear risk.

The Howley et al study was automatically excluded from the forest plot as there are zero events in both arms, we left it only in the table to show that zero events are not estimable

In the periapical radiolucency outcome the two trials included were Howley et al. and Aminabadi et al, , the study of Nguyen et al. was excluded due to lack of values in this outcome, only odds ratio was reported with no prevalence percentage in the control or non exposure group so we could not convert the odds ratio to risk ratio.

Lastly in the conclusion section:

Radiographic assessment was added.

# Peer review: Reviewer response for version 1

**DOI:** 10.5256/f1000research.17628.r41014

**Published:** 2018-12-03

**Authors:** Armin Shirvani

**Affiliations:** 1 Iranian Center for Endodontic Research, Research Institute of Dental Sciences, Faculty of Medical Education, Shahid Beheshti University of Medical Sciences, Tehran, Iran

The manuscript is well structured and the format of the report on the details of the study is acceptable.

 There are two points to consider in the Meta-analysis:

1. The results of Hawley’s study should not have been removed because of zero in nominators. They could use 0.01 instead of 0 and continue the analysis.
2. Heterogeneity in time intervals is an important issue and if a significant heterogeneity has been detected the results of the meta-analysis would not be valid.

I confirm that I have read this submission and believe that I have an appropriate level of expertise to confirm that it is of an acceptable scientific standard, however I have significant reservations, as outlined above.

# Response

**Published:** 2018-12-09

**Authors:** Lamia Gadallah

**Affiliations:** National Research Centre, Egypt, Egypt

Dear Dr. Armin, 

Many thanks for reviewing our study and for your valuable comments.

Concerning your first comment on the exclusion of Howley et al. study from the clinical failure meta-analysis, we returned to Cochrane handbook for systematic reviews of "intervention that stated that". The standard practice in meta-analysis of odds ratios and risk ratios is to exclude studies from meta-analysis where there are no events in both arms. This is because such studies do not provide any indication of either the direction or magnitude of the relative treatment effect"

Actually the Revman program that we used for meta-analysis does not accept any decimals in the events in risk ratio as dichotomous outcomes.

As for the heterogeneity in the radiographic evaluation, we performed a sensitivity analysis and it was due to one outlying study so we performed the meta-analysis both with and without this study and we stated that the results should be interpreted with caution.

# Peer review: Reviewer response for version 1

**DOI:** 10.5256/f1000research.17628.r39650

**Published:** 2018-11-07

**Authors:** Mariem O. Wassel

**Affiliations:** 1 Department of Pediatric Dentistry and Dental Public Health, Faculty of Dentistry, Ain Shams University, Cairo, Egypt

The systematic review and meta-analysis regarding pulpotomy versus pulpectomy in the treatment of vital pulp exposure in primary incisors is well designed and that the methodology  and discussion are well written. One question I have is: was the protocol of this review registered before carrying out the research? Some mistakes in writing the manuscript are present which are:

- Please be sure the reference number is written right after the author name through-out the whole manuscript.
- In the background section of the abstract: This study aimed to assess the (effectiveness) of pulpotomy and pulpecomy (pulpectomy) in treatment of carious vital pulp exposure in primary incisors.
- In the conclusion section of the abstract; Further high quality studies comparing between pulpotomy and pulpectomy in primary incisors with longer follow up period till exfoliation time    (are needed).
- Page 2, introduction section:
  1. In pulpectomy(,) a resorbable material such as nonreinforced
  2.  According to a Cochrane systematic review(,) there was no
  3. Endoflas were found to be equally effective(.) (W)hile(,) with (a) low quality of evidence(,) zinc oxide and eugenol may be better than
  4. We(,) therefore(,) aimed to determine in patients with carious vital pulp exposure in primary incisors(,) if pulpotomy is better than

 Page 6:

- Duration of follow up.:
- “Follow up was at 12 and 24 months in two trials, by Aminabadi *et al[*ref-1] and Casas *et al* ^2^.” .do you mean that both studies used a 12 and 24 months  follow up period or that the first study used 12 months follow up while the second was 24 months? Please clarify.
- Follow up was (up) to 23 months at three intervals: 5–9, 10–14, and 15–23 months
- Anesthesia: under local anesthesia (by)  Aminabadi *et al* ^1^ *. *
- Medicaments -- Pulpotomy: please rewrite this paragraph noting that 2 trials performed formocresol pulpotomy as we don’t achieve hemostasis with formocresol.
- Pulp access: In the trial (by) Howley *et al* ^3^ the pulp chamber was unroofed using
- Pulpotomy: in another (the other)  two trials by Nguyen *et al[*ref-4] and Casas *et al[*ref-2].
- Final restoration : Resin restorations was  (were) performed in three….. Full coverage crowns whether stainless steel crown (SCC) or SCC with white esthetic veneer( were used ) in Howley *et al( trial )* ^3^.
- Results of studies;
- nor pulpectomy in this (the) 23 month(s)  trial by Howley et al ^3^. The clinical failure rate was 13.1% for pulpotomy and 4.4% (for pulpectomy) at

 page 7:  

- radiological failure.: was in (remove "in")  41% in the pulpotomy group and 18% in pulpectomy group at 2 years follow up trial by Casas *et al[*ref-2].
- Pain: No pain was reported in either group(s) in Howley *et al* ^3^ *. *trial9.  
- No pain was reported in either group(s) in the Casas *et al* ^2^ *. *trial21.
- Soft tissue pathology: No soft tissue pathology was reported in either group(s) in Howley et al ^3^. (trial)9.  
- Pathological mobility: Pathologic mobility was not reported for any tooth in three trials (;)  Howley et al ^3^.9(,) ,Aminabadi et al ^1^.20 and Casas et al ^2^.21.

 Pathological radiolucency:

- At 2 years follow up(,) 5 teeth (11.11%) showed
- follow up(,) 7 teeth (58%) showed periapical radiolucency in the  
- pathological root resorption: while for internal resorption(,) one tooth (3%) showed perforating  
- At 2 years follow up(,) pathologic external or internal root resorption occurred in 6 teeth (13.3%)  (in) the pulpotomy group and in 2 teeth (4.34%)  (in) the pulpectomy group in the Aminabadi *et al. *trial20
- At 2 years follow up(,) pathologic external root resorption occurred  

Pulp canal obliteration:

- At 23 months(,) pulp canal obliterationmwas seen in 18 teeth (60%) in the pulpotomy group in Howley *et al.*9 (trial). At 2 year follow up(,) no teeth showed pulp canal obliteration

 Pages 8 and 9:

- Please add reference number to authors names in the titles of tables 4 to 7.

Page 11, discussion section:

- 2 ^nd^ paragraph:  After exclusion of one trial due to its high risk of bias(,) (only) three trials (were left)  to be included in the meta-analysis.(were left) to be included in the meta-analysis.
- Overall failure was reported for two pulpotomized incisors in two trials. (on the other hand,)   tooth survival(,) although it is an important outcome(,) but it is not commonly reported.
- The overall risk of bias of primary studies was low for three trials, except for the unclear risk for blinding of clinical assessment which was not effective(.)(W)e did not have access to all the trial protocols to assess the selective reporting bias except (for) only one trial.

I confirm that I have read this submission and believe that I have an appropriate level of expertise to confirm that it is of an acceptable scientific standard.

## References

[1] : A clinical study of formocresol pulpotomy versus root canal therapy of vital primary incisors. *J Clin Pediatr Dent*.2008;32(3) :211-4 1852427110.17796/jcpd.32.3.ghk26v4554790074

[2] : Outcomes of vital primary incisor ferric sulfate pulpotomy and root canal therapy. *J Can Dent Assoc*.2004;70(1) :34-8 14709254

[3] : Pulpotomy versus pulpectomy for carious vital primary incisors: randomized controlled trial. *Pediatr Dent*.34(5) :112-9 23211895

[4] : Comparison of Ferric Sulfate Combined Mineral Trioxide Aggregate Pulpotomy and Zinc Oxide Eugenol Pulpectomy of Primary Maxillary Incisors: An 18-month Randomized, Controlled Trial. *Pediatr Dent*.2017;39(1) :34-38 28292339

# Response

**Published:** 2018-11-08

**Authors:** Lamia Gadallah

**Affiliations:** National Research Centre, Egypt, Egypt

Thank you Dr. Mariem very much for reviewing our study. As for the protocol registration, yes it was registered in the Evidence based Committee of Cairo University as the preliminary step of my Ph.D. in 2014.
